# Community-Engaged Approach to Increase Physical Activity Among Black Individuals With Colorectal Cancer: Protocol for a Feasibility Randomized Controlled Trial of the Physical Activity Centers Empowerment Study

**DOI:** 10.2196/65804

**Published:** 2025-10-17

**Authors:** Rachel Hirschey, Jingle Xu, Nathaniel Woodard, Paulette Duggins, Deirdre F Lea, John L Milner, Karia Coleman Jr, Ashley Leak Bryant, Hanna K Sanoff, Tammy Triglianos, Baiming Zou, Natasha Renee Burse, Rebecca L Hoover, Jennifer Leeman, Stephanie B Wheeler, Claudio L Battaglini, Carmina G Valle

**Affiliations:** 1 School of Nursing University of North Carolina at Chapel Hill Chapel Hill, NC United States; 2 Lineberger Comprehensive Cancer Center University of North Carolina at Chapel Hill Chapel Hill, NC United States; 3 Department of Community Health and Health Behavior, School of Public Health and Health Professions University at Buffalo Buffalo, NY United States; 4 Stiving to Hold Accountability in Research Equity Community Advisory Board University of North Carolina at Chapel Hill Chapel Hill, NC United States; 5 Division of Oncology, Department of Medicine University of North Carolina at Chapel Hill, School of Medicine Chapel Hill, NC United States; 6 Department of Biostatistics, Gillings School of Public Health University of North Carolina at Chapel Hill Chapel Hill, NC United States; 7 Mark and Robyn Jones College of Nursing Montana State University Bozeman, MT United States; 8 Department of Health Policy and Management, Gillings School of Public Health University of North Carolina at Chapel Hill Chapel Hill, NC United States; 9 Department of Exercise and Sport Science University of North Carolina at Chapel Hill Chapel Hill, NC United States; 10 Department of Nutrition, Gillings School of Global Public Health University of North Carolina at Chapel Hill Chapel Hill, NC United States

**Keywords:** African American, community-based participatory research, physical activity, colorectal cancer, disparities

## Abstract

**Background:**

Black individuals are more likely to die from colorectal cancer (CRC) and experience more treatment-related side effects compared to White individuals. Physical activity (PA) has been associated with decreased side effects, improved CRC treatment completion rates and responses, and survival. However, Black survivors of CRC are 60% less likely to engage in PA than White survivors. The Physical Activity Centers Empowerment (PACE) study is testing an intervention specifically designed to increase PA among Black individuals diagnosed with CRC.

**Objective:**

This study outlines the protocol for a randomized controlled trial. The study aims to test the feasibility of PACE and will use the reach, effectiveness, adoption, implementation, and maintenance (RE-AIM) framework.

**Methods:**

The PACE study was developed in partnership with a community advisory board consisting of Black cancer advocates and survivors of cancer. The study aims to recruit 72 participants aged >18 years from North Carolina who have been diagnosed with CRC. These participants will be randomized in a 1:1 ratio to an intervention or control group. During the 12-week intervention, all participants will receive a wearable activity tracker and informational materials from the American College of Sports Medicine’s “Moving through Cancer” program. The intervention group will also receive additional PACE theory–guided intervention components, including personalized daily adaptive step goals, access to the PACE video library, and optional video chat meetings for PA support. Data will be collected at 3 time points: baseline, after the intervention (3 months), and 6 months after the intervention (9 months). Using the RE-AIM framework, the study aims to evaluate the intervention’s reach, effectiveness, acceptability, implementation, and maintenance.

**Results:**

The National Institute on Minority Health and Health Disparities funded this study in 2021. Study enrollment began in August 2024 and is anticipated to conclude in December 2024.

**Conclusions:**

This study will advance our understanding of effective behavioral strategies to increase PA and help advance the use of PA as a form of complementary cancer treatment, with the aim of improving health outcomes for Black survivors of CRC.

**Trial Registration:**

ClinicalTrials.gov NCT06411756; https://clinicaltrials.gov/study/NCT06411756

**International Registered Report Identifier (IRRID):**

DERR1-10.2196/65804

## Introduction

### Background

Colorectal cancer (CRC) has a disproportionate impact on Black individuals, who experience higher incidence rates and CRC-related mortality compared to White individuals [1]. Black Americans also experience a higher CRC symptom burden compared to White individuals, including treatment-related side effects, such as pain, fatigue, depression, and bowel dysfunction, which affect their quality of life [2]. Physical activity (PA) has been shown to help manage these side effects, improve treatment completion rates and responses, and improve survival [3]. Despite the safety and benefits of PA during CRC treatment [4,5], Black survivors are 60% less likely to engage in PA compared to White survivors [6]. This disparity underscores the urgent need for targeted, culturally relevant interventions that promote PA during CRC treatment in Black populations. However, few interventions have been designed specifically with and for this population, and even fewer have been evaluated for feasibility before scale-up.

The Physical Activity Centers Empowerment (PACE) study tests a PA intervention designed with and for Black survivors of CRC. Grounded in the National Institute for Minority Health and Health Disparities (NIMHD) Research Framework [7] and the affective-reflective theory (ART) of physical inactivity and exercise [8], the intervention was developed through qualitative research with Black survivors of cancer and oncology nurses, identifying key barriers and facilitators to PA engagement. This foundational work informed both the structure and content of the PACE intervention, ensuring alignment with community needs and evidence-based behavior change strategies. This study aims to assess the feasibility of the PACE intervention. Measurement of feasibility will be based on applying the reach, effectiveness, adoption, implementation, and maintenance (RE-AIM) planning and evaluation framework to the intervention [9].

A feasibility study is a critical and appropriate next step before launching a fully powered trial. It allows the evaluation of core implementation outcomes, including recruitment, retention, intervention delivery, and participant engagement, each of which is essential to understanding whether the intervention is acceptable, practical, and sustainable in real-world settings. This is particularly important when working with populations considered historically underserved, such as Black survivors of CRC, where community engagement and contextual fit must be rigorously assessed before moving to effectiveness trials.

The PACE study was created and is being implemented and disseminated in collaboration with the members (PD, DFL, JLM, and KC) of Stiving to Hold Accountability in Research Equity (SHARE)—a community advisory board whose mission is to work with researchers to identify community needs and promote evidence-based interventions to reduce cancer disparities that impact Black and African American communities [10]. The PACE study protocol is detailed in the study, with our reporting guided by the Standard Protocol Items: Recommendations for Interventional Trials (SPIRIT) checklist—a guide for reporting clinical trial protocols. (Multimedia Appendix 1). Recruitment of participants began in August 2024 following version 1 of the study protocol.

### Objectives

The objective of this study is to describe the design, rationale, and protocol of the PACE feasibility trial. The PACE trial will evaluate the feasibility of delivering a culturally tailored, theory-based PA intervention among Black survivors of CRC and will generate preliminary data on implementation and potential intervention impact. The findings from this study will inform future efficacy and dissemination efforts.

## Methods

### Study Design

**Figure 1:**
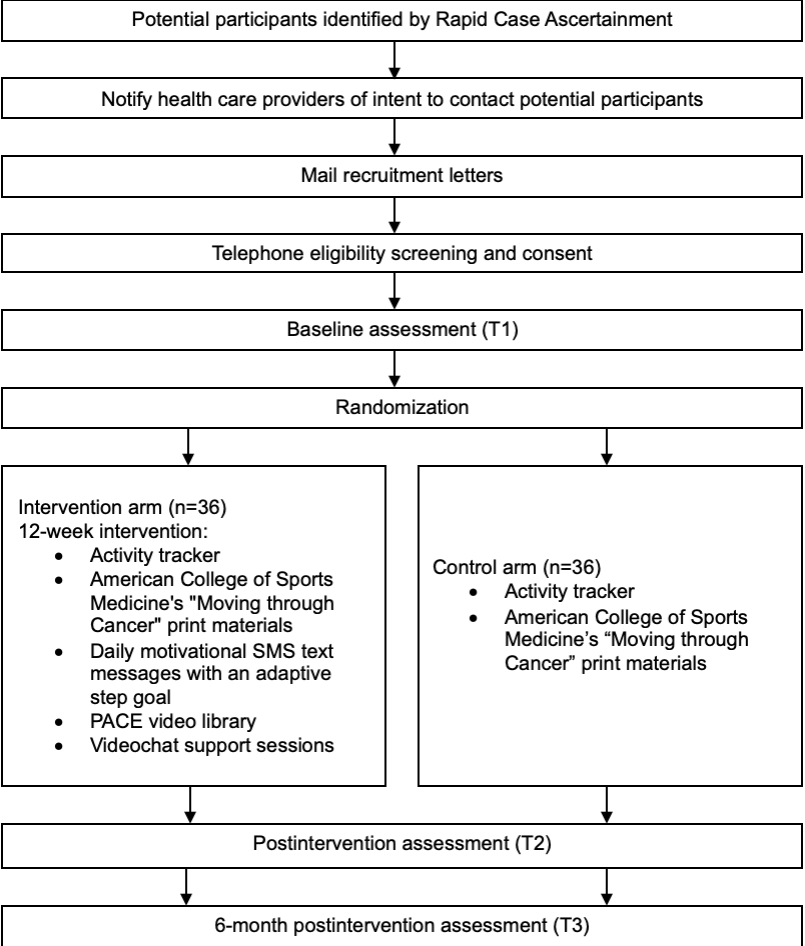
Overview of the study flow for the Physical Activity Centers Empowerment (PACE) study.

The PACE feasibility study is a community-based 9-month 2-arm parallel group randomized controlled trial. A total of 72 participants will be enrolled and randomized 1:1 to either an intervention group or a control group. All participants will receive a wearable activity tracker (Fitbit Inspire, Fitbit, Inc) and the American College of Sports Medicine’s “Moving Through Cancer” materials [11]; intervention arm participants will receive the 12-week PACE intervention. Participants will complete study measures at baseline, immediately after the intervention (3 months after baseline), and 6 months after the intervention. Participation will be discontinued if a participant dies or requests to withdraw from the study. Figure 1 shows an overview of the study flow.

### Ethical Considerations

This study was approved by the University of North Carolina (UNC) Lineberger Comprehensive Cancer Center Protocol Review Committee and the UNC Chapel Hill Institutional Review Board (IRB; 21-0881). The PACE feasibility trial is registered with ClinicalTrials.gov (NCT06411756).

### Conceptual Framework

**Figure 2:**
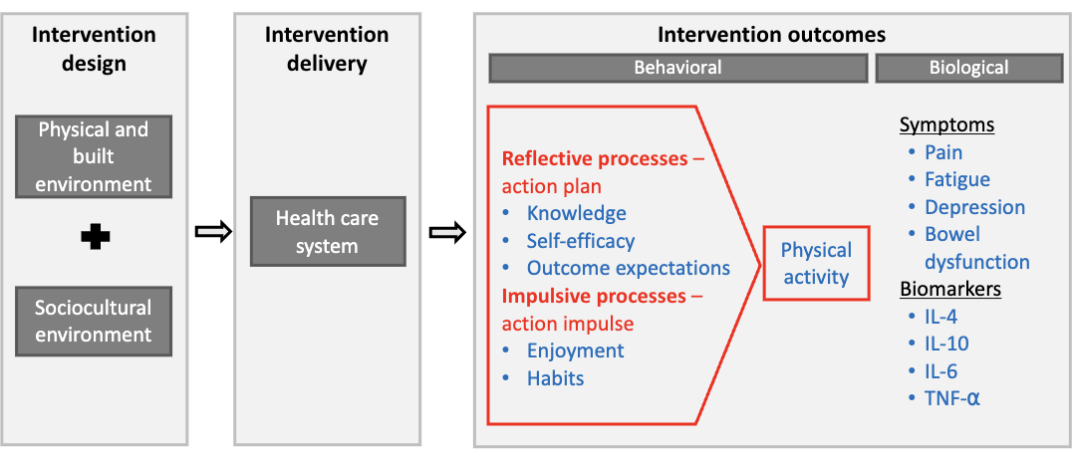
Physical Activity Centers Empowerment guiding framework. Text in gray boxes indicates the National Institute for Minority Health and Health Disparities domains and how they structure the study. Text in red outlines the study’s theoretical framework. Text in blue indicates the outcome variables measured. IL: interleukin; TNFα: tumor necrosis factor α.

The conceptual framework guiding PACE (Figure 2) follows the NIMHD research framework’s 5 domains of health influence (ie, built and physical environment, sociocultural environment, health care system, behavioral outcomes, and biological outcomes) [7]. To develop the PACE framework, the aforementioned qualitative study [12] was conducted with Black survivors of cancer to identify PA barriers and facilitators related to the physical and built and sociocultural environment domains. In addition, a survey was conducted with oncology nurses to evaluate the feasibility of delivering the intervention within the health care system. Findings showed that while nurses recognized the benefits of PA interventions for patients, implementation in clinical practice was challenging because of competing demands and limited resources [13]. These findings align with a systematic review on nurse recommendations for PA during CRC treatment, which concluded that while providing PA recommendations is feasible for nurses, integrating a full PA intervention into clinical practice is not [14]. Findings from these formative studies with Black survivors of cancer and oncology nurses were applied to develop the PACE intervention, which aims to improve PA and related outcomes in the behavioral domain, which, in turn, are hypothesized to improve outcomes in the biological domain, including inflammatory biomarkers, symptoms, and side effects.

### Intervention Overview

The PACE intervention was informed by qualitative research conducted with SHARE, which highlighted the need for a PA intervention for Black individuals undergoing cancer treatment to address physical and psychological challenges; incorporate support from faith, family, other survivors, and the Black community; emphasize psychological benefits of PA; and include realistic, flexible goals [13]. On the basis of these insights, potential intervention components were drafted and refined through qualitative interviews with 9 Black survivors of CRC. The study team and SHARE members reviewed the findings to finalize the intervention. The key design elements determined from these interviews include Fitbit use, SMS text messages emphasizing PA’s mental health benefits, individualized step goals, personal story videos, PA resources, side effect management strategies, and print materials.

The PACE intervention is guided by the ART of physical inactivity and exercise, which accounts for both reflective processes and impulsive processes that influence PA behavior [8]. In contrast, most behavioral theories commonly used in PA interventions focus solely on reflective processes, predicting PA intentions but having limited impact on PA behavior. Thus, the PACE intervention targets theoretical constructs that relate to reflective (ie, knowledge, self-efficacy, and outcome expectations) and impulsive processes (ie, enjoyment and habit). Structured around these constructs, behavior change techniques (BCTs) [15] are applied in the intervention to increase PA. Multimedia Appendix 2 provides an overview of intervention components, theoretical constructs targeted, and BCTs used in each component.

### Community Engagement

PACE recruitment methods were created in collaboration with the Angelic Warrior Foundation (AWF). The AWF is a nonprofit organization based in North Carolina focused on raising CRC awareness and supporting patients with CRC and their families (Angelic Warrior Foundation, Inc. 2020). The PACE researchers and AWF work closely on research and community CRC awareness and support initiatives, with the AWF president (PD) serving as a SHARE member (ie, the community advisory board for PACE) and the PACE principal investigator (RH) serving on the AWF board of directors. AWF played a key role in shaping recruitment strategies by drafting study introduction letters, codeveloping all materials included in recruitment packages, and collaborating on the timing and frequency of participant outreach via mail and telephone.

### Recruitment

Potential study participants will be identified through the Rapid Case Ascertainment (RCA) program. The RCA program is a collaboration between the North Carolina Central Cancer Registry and the Lineberger Comprehensive Cancer Center at UNC Chapel Hill, designed to rapidly identify newly diagnosed cancer cases and facilitate timely recruitment for research studies. Individuals diagnosed with CRC in the past year will be identified through the RCA program, which will provide the PACE team with the contact information for both the individuals and the physicians who diagnosed their cancer. Following RCA protocols, the PACE team will first contact each provider for permission before mailing study information to the potential participants.

### Eligibility Criteria and Screening

One week after the recruitment package is mailed, study staff will call potentially eligible participants to assess their interest in study participation and confirm eligibility among those interested. Inclusion and exclusion criteria are listed in Textbox 1. The Physical Activity Readiness Questionnaire (PAR-Q) [16] will be used to assess participant safety for engaging in PA. Per PAR-Q guidelines, individuals who endorse items 1 to 4 (having a diagnosed heart condition requiring doctor-recommended PA, experiencing chest pain during PA, recent chest pain, or episodes of dizziness or loss of consciousness) will be ineligible to participate. Individuals endorsing items 5 to 7 (bone or joint problems aggravated by PA, use of prescription medications for blood pressure or heart conditions, or other medical concerns) will be eligible if they obtain provider approval. In addition, we acknowledge the availability of the updated PAR-Q for Everyone and will consider its use in future studies. Potential participants confirmed to meet the eligibility criteria will complete verbal informed consent and a demographic survey over the telephone. Consenting participants will be mailed a data collection package that includes materials for blood self-collection, a prepaid return package to return blood samples, and the wearable activity tracker with setup instructions. Participants will be sent video instructions on setting up and using the activity tracker (Fitbit Inspire) and completing self-collection blood samples. Participants will be provided a link to video instructions for blood collection. Consenting participants will also receive a unique URL to complete baseline survey measures on the web through a secure web-based data capture program called REDCap (Research Electronic Data Capture; Vanderbilt University) [17]. Participants will be emailed an authentication web link to authorize the study team to access data from their activity tracker. Within a week of the activity tracker being mailed, study staff will assess whether the participant’s data from the device are accessible. If a participant’s data are not accessible, staff will contact the participant and follow up daily for 7 days, as needed, to troubleshoot the activity tracker setup process.

**Inclusion and exclusion criteria for participants in the Physical Activity Centers Empowerment (PACE) study.**

**Inclusion criteria**

- Self-identify as African American or Black
- Histological diagnosis of adenocarcinoma, goblet cell, adenosquamous, or medullary carcinoma of the colon or rectum
- Aged >18 years

**Exclusion criteria**

- Endorsing items 1 to 4 on the Physical Activity Readiness Questionnaire (PAR-Q; ie, being told by a doctor that you have a heart condition and should only do physical activity [PA] recommended by a doctor, experiencing chest pain when doing PA, chest pain within the last month, or loss of balance because of dizziness or losing consciousness)
- Endorsing items 5 to 7 on the PAR-Q (ie, bone or joint problems that could be worsened by PA, currently taking prescription drugs for blood pressure or a heart condition, or other known reasons you should not do PA) and failure to obtain provider consent for participation
- Plans for a major surgery other than colorectal cancer–related surgery within the next 6 months (eg, knee replacement)
- Current participation in another PA research study
- Pregnant or planning to become pregnant
- Does not own a smartphone with active data plan (required for synchronizing with the wearable activity tracker)
- No internet access (required for viewing PACE videos)
- Not willing to be randomly assigned
- Not willing to use the wearable activity tracker throughout the study

### Randomization

After confirmation that baseline surveys have been completed, a research assistant will enroll participants using a random number list generated in REDCap tools to assign participants to the intervention or control group in a 1:1 ratio. The tool conceals the sequence until the research assistant clicks the button to complete the randomization. Participants will not be aware of their randomization results. However, participants will be able to self-identify whether they receive the PA intervention. Study arm assignments will be revealed only to research assistants responsible for mailing study materials to ensure the correct materials are mailed. In addition, those who lead monthly video chat meetings will identify that the meeting participants are assigned to the intervention arm.

### Interventions

#### Control Arm

Control arm participants will receive an activity tracker and the American College of Sports Medicine’s Moving through Cancer” booklet. These components were selected to create a control condition that provides widely available and commonly used activity trackers and publicly available PA recommendations. The control condition will facilitate the examination of the effects of the theory-guided PACE intervention components.

#### Intervention Arm

In addition to the activity tracker and booklet, intervention arm participants will also receive (1) a daily adaptive step goal via an SMS text message with a short positive message, (2) access to the PACE video library, and (3) optional monthly video chat (Zoom) meetings for PA support.

#### Daily Adaptive Step Goal Sent With a Short Positive Text Message

**Figure 3:**
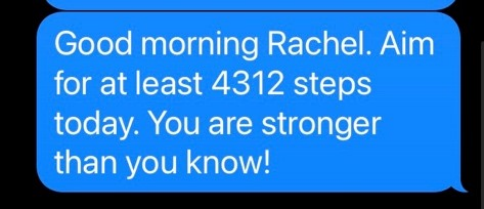
Example of a daily SMS text message.

Participants will be asked to wear their PACE-issued activity tracker daily throughout the PACE intervention. The tracker will display the number of steps a participant takes throughout a given day. The study team will collect daily steps from participants’ accounts associated with the tracker, which will be used to generate a tailored goal for each participant that is responsive to their changing PA levels as they navigate cancer and treatment side effects. Using a previously developed rank order algorithm [18,19], daily step goals will be set at the 60th percentile of participants’ daily step counts from the past 7 days. Daily step goals will be texted to participants each morning with a short positive message; Figure 3 shows an example SMS text message.

#### PACE Video Library

Throughout the 12-week intervention, web links for each of the 22 PACE videos will be sent to participants via SMS text messaging and email. Once a video is released, participants can view it as many times as they wish for the duration of the study. Each video was created through an interactive process between PACE researchers and SHARE members. Researchers created a first draft for each video topic that included references to exercise oncology and behavior change–related evidence. The topics were selected based on a literature review of PA relevant for survivors of cancer, combined with topics identified in our formative qualitative work and input from SHARE members. Messages within each video address ART constructs (ie, knowledge, self-efficacy, outcome expectations, habits, and enjoyment) and include BCTs [15], as detailed in Multimedia Appendix 2. According to the narrative theory [20], information is processed more deeply, believed, and acted upon when messages are from individuals with whom one identifies. Thus, SHARE members, who are Black survivors of cancer and caregivers, narrated the videos. The respective narrator edited each video script to include their personal experiences and voice. Next, a draft of each script was read, reviewed, and edited in real time during an in-person or virtual meeting between a researcher and SHARE members. The researchers then reviewed, edited, and coded the scripts for BCT content. Finally, the respective narrator for each script provided a final review and edits to ensure the messaging felt like it was in their voice before recording.

#### Optional Monthly Video Chat Meetings for PA Support

The virtual PA support meetings will be delivered via Zoom video conferencing software [21] and facilitated by a researcher following the focus group methodology [22]. One member of the SHARE community advisory board will attend each session. Each meeting will begin with a confidentiality review, during which all members will be asked to ensure they are in a private place where others cannot hear or see the meeting. All members will verbally state that they commit to keeping the identities and experiences of other group members confidential. The SHARE member will begin with a welcome and group introduction, and then, the researcher will ask questions to guide conversations about each of the theoretical constructs of the PACE study (ie, knowledge, self-efficacy, outcome expectations, habits, and enjoyment). The SHARE member will provide a closing and thank participants for attending the meeting. Meetings will not be recorded; however, the researcher will take notes to capture the main themes of the conversation. The researcher will also document the attendance of participants in a secure university-sponsored and IRB-approved digital folder.

### Data Collection

All outcomes will be measured at baseline, immediately after the intervention (3 months after baseline), and 6 months after the intervention (9 months after baseline). For each assessment point, survey links will be sent via email and SMS text messaging; up to 4 reminders to complete surveys will be sent. Participants may begin the surveys and return to complete them later using a link provided by email upon request. Participants will be sent blood self-collection kits and a prepaid return package at each time point. The collection materials will be labeled with a biosample identifier number unique from their study identifier. The return package will be preaddressed and will contain the return address of the PACE research study to protect against the risk of samples being misplaced or participants being identified during the mailing process. No identifying information beyond the blood samples will be included in the blood return package. If the research team does not receive blood kit return packages within 2 weeks of being mailed, study staff will call and email participants for a maximum of 3 attempts to support participants in completing blood sample collections. If a participant’s daily step count is <200 for 4 consecutive days, an automated email will remind them to wear their activity tracker. Study staff will contact participants via telephone and email for a maximum of 3 attempts to troubleshoot their activity tracker use. Participants in the intervention arm will complete a 15-minute telephone interview after the intervention that includes three questions: (1) What aspects of the PACE intervention did you find most beneficial? (2) Were there any challenges or difficulties you faced while participating in the intervention? (3) Do you have any suggestions for improving the PACE intervention?

### Outcome Measures

#### Feasibility

The primary study outcome, feasibility, will be determined using the RE-AIM framework [11], with each of the 5 key RE-AIM elements assessed independently and through specific criteria. Rather than a simple “yes or no” determination or a composite score, feasibility will be evaluated based on thresholds for each element. Specifically, reach will be tested by measuring the number of participants contacted, found eligible, recruited, and retained for the duration of the study. On the basis of enrollment in other similar studies [16,23] involving Black participants and patients with CRC, we determined that feasibility would be achieved if 72 participants are enrolled within 18 months and the total attrition rate is <20%. Intervention effectiveness will be tested by measuring intervention effects on selected behavioral, psychosocial, and health-related outcomes, as well as inflammation biomarkers, as detailed subsequently. We anticipated that, compared to the control group, the intervention group will have higher scores on behavioral, psychosocial, and health-related outcomes, as well as on anti-inflammatory biomarkers, and lower scores on proinflammatory biomarkers. Adoption will be measured as the percentage of providers who agree or grant consent for their patients to participate. Implementation fidelity will be assessed as the extent to which the intervention is delivered as intended. This will be measured by the percentage of intervention days that participants wear their Fitbits, the percentage of eligible intervention arm participants attending support sessions, and the percentage of videos viewed relative to the total number of available videos across all participants. While limited published guidance exists on a priori feasibility thresholds [24,25], we have set the feasibility threshold at 60%. This decision is based on recommendations for pilot studies, which suggest selecting a threshold that allows identifying areas where adaptations and improvements can be made in a subsequent efficacy trial [26]. Intervention uptake will be assessed through participant interviews. Intervention arm participants will complete a 15-minute interview via Zoom after the intervention. Using a semistructured format, participants will be asked about their experiences participating in the PACE intervention. Maintenance will be assessed by measuring intervention effects on selected behavioral, psychosocial, health-related outcomes, and inflammation biomarkers 6 months after the intervention. We anticipated that compared to the control group, the intervention group will have higher scores on behavioral, psychosocial, and health-related outcomes, as well as on anti-inflammatory biomarkers, and lower scores on proinflammatory biomarkers.

#### PA Measures

PA will be measured with wearable activity trackers (ie, Fitbit Inspire), which will capture daily minutes of vigorous, moderate, and light exercises; sedentary time; and the number of daily steps. The data will be used to calculate weekly steps; weekly minutes of light-, moderate-, and vigorous-intensity PA; active days per week; and weekly sedentary minutes. The wearable activity trackers used in this study have demonstrated satisfactory validity on step counts measured by metabolic equivalents [27]. PA will be measured subjectively using an adapted Godin Leisure-Time Exercise Questionnaire [28,29], which asks participants to self-report their weekly minutes of strenuous, moderate, and mild or light exercise. The Godin Leisure-Time Exercise Questionnaire demonstrates good reliability and validity compared to other self-report PA measures [30,31] and has a test-retest reliability coefficient of 0.94, 0.46, and 0.48 for self-reported strenuous, moderate, and light exercises, respectively [32]. Fitbit data will serve as the primary measure of PA. Objective and subjective PA measures will be analyzed in parallel and compared. Where discrepancies arise, descriptive analyses and sensitivity tests will be conducted to explore sources of variation.

This feasibility pilot study is not powered to detect statistical significance. On the basis of prior longitudinal PA intervention studies for survivors of cancer [33-35], conservatively, we anticipate 17% total attrition. Therefore, we will enroll 72 participants to obtain a final sample of 60 (30 in each group). With 30 participants per group, we will have 80% power to detect a standardized mean difference of *d*=0.74 at 9 months, which is a large effect for a PA intervention.

#### Sedentary Behaviors

Sedentary behaviors will be measured using the Sedentary Behavior Questionnaire [36], which includes 9 common sedentary behaviors and measures the number of hours per day participants spend doing sedentary behaviors. The questionnaire has shown moderate to excellent intraclass correlation coefficients (0.48-0.93) for the time spent on each sedentary behavior and the total sedentary time and acceptable validity for measuring sitting and inactivity minutes [36].

#### Psychosocial Constructs

Psychosocial constructs will be measured using previously validated scales. PA knowledge will be measured using a behavioral capacity measure primarily developed by Short et al [37]. This measure is a 5-point Likert scale assessing participants’ knowledge about the types, intensity, frequency, and skills of doing PA [37]. Self-efficacy will be measured using a 9-item barrier self-efficacy scale and a 4-item task self-efficacy scale, which assess participants’ confidence in overcoming barriers to PA and their ability to engage in PA. The 2 self-efficacy scales have Cronbach α values of 0.96 and 0.89, respectively, among patients with cancer [38]. Outcome expectations will be measured using the 19-item Multidimensional Outcome Expectations for Exercise Scale, which has an internal consistency of 0.82, 0.84, and 0.81 for physical, self-evaluative, and social outcome expectations, respectively [39]. PA habits will be measured using a 6-point bipolar scale that assesses the 4 indicators of habit strength:: negative consequences if not done, patterned action, stimulus-response solid bonds, and automaticity [40]. The scale has Cronbach α values ranging from 0.77 to 0.85 for the 4 PA habit indicators [40]. Finally, the 4-item short version of the Physical Activity Enjoyment Scale will measure the enjoyment of doing PA [41]. This scale has internal consistency values ranging from 0.82 to 0.87 among diverse populations.

#### Patient-Reported Outcomes

Patient-reported health outcomes—commonly observed among patients with CRC and known to be positively influenced by PA—will be measured [42]. These include pain, fatigue, depression, bowel dysfunction (ie, diarrhea and constipation), quality of life, and sleep. These outcomes will be measured using the Patient-Reported Outcomes Measurement Information System (PROMIS) tools [43]. The PROMIS pain, fatigue, depression, and sleep measures have reliability coefficients of 0.96, 0.94, 0.91, and 0.88, respectively, with excellent validity among survivors of cancer [44,45]. The diarrhea and constipation scales also have internal reliability values of 0.88 and 0.89, respectively [46]. Neurotoxicity will be measured using the Functional Assessment of Cancer Therapy/Gynecologic Oncology Group–Neurotoxicity 4 scale. The internal consistency of this scale ranges from 0.73 to 0.91 at different assessment cycles among patients with cancer [47]. Quality of life will be measured using the 36-item Short Form Health Survey [48], which is a widely used survey for assessing respondents’ general physical and mental health status to determine their quality of life among survivors of CRC [49], with an acceptable to high internal consistency (>0.07) and validity observed among survivors of cancer [50,51].

#### Inflammation Biomarkers

Inflammation biomarkers that are thought to mediate the effects of PA on cancer mortality will be measured [52,53]. These include interleukin (IL)-4, IL-10, IL-6, and tumor necrosis factor α and will be measured using serum samples collected with a dried blood spot (DBS) card kit [54]. DBS cards are widely used to assess biomarkers, such as antibodies [55]. The validity of using the DBS card to measure these inflammation biomarkers in this sample will be evaluated with a comparison to whole blood samples collected using a Tasso+ kit [56]. Although DBS cards have been validated in previous studies, we will also validate them with this sample. Because the purpose of this testing is to assess the validity of DBS cards in accurately measuring the biomarkers of interest, a convenience sample is appropriate. Therefore, we will recruit the first 10 consenting participants to ensure we collect the necessary samples. Participation in this part of the study is optional and will not impact study inclusion.

### Retention

**Figure 4:**
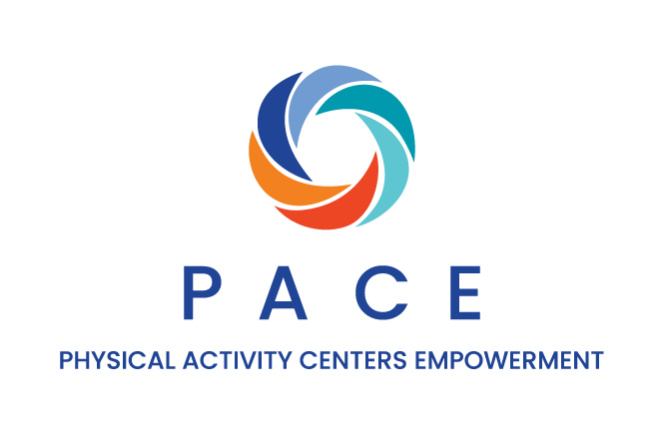
Physical Activity Centers Empowerment (PACE) logo.

To support participants’ adherence to the study and build their understanding of the importance and value of their participation in the PACE study, we include the PACE logo (Figure 4) in all study documents to help create a sense of identity and the PACE study community. Participants will receive monthly PACE study update cards with a message thanking them for their time; a statement about what the study team hopes to learn from their participation; plans for community and scientific dissemination of findings; and a positive affirmation note from the AWF, our community partner organization described earlier. Participants will receive a US $30 gift card for completing the baseline assessment, a US $40 gift card for completing the 3-month assessment, and a US $50 gift card for completing the 9-month assessment. Intervention arm participants who complete interviews will receive an additional US $15 gift card. The 10 participants who complete the Tasso blood collections will receive an additional US $10 gift card.

### Safety and Monitoring

At weekly PACE research team meetings, all issues relevant to study progress, including but not limited to enrollment, safety, regulatory, and data collection, will be reviewed. Unanticipated adverse events will be reported to the IRB within 1 week. Study documents will be made available for inspection when requested by any of the regulatory bodies charged with the safety of human participants and the integrity of data, including, but not limited to, the Office of Human Research Ethics IRB, the Oncology Protocol Review Committee, and the North Carolina Translational and Clinical Sciences Institute Data and Safety Monitoring Board.

Potential participants’ information from RCA, including identifiable information (eg, names, phone numbers, and physical addresses), will be stored on a secure university server and will only be available to members of the research team. Identifiable information for potential participants who do not enroll in the study will be removed when the RCA recruitment is completed. For those who enroll, identifiable information will be removed at the study’s completion unless participants have agreed to be contacted by the research team for future studies.

### Statistical Data Analysis Plans

#### Reach

Descriptive statistics will be used to calculate the number of participants contacted and the percentage eligible, recruited, and retained for the duration of the study.

#### Adoption

Descriptive statistics will be used to calculate the percentage of providers who agree to have their patients contacted about the study out of the total number approached, as well as the percentage of providers who grant consent for their patients to participate in PACE out of the total number asked for consent based on the PAR-Q screener.

#### Implementation Fidelity

Descriptive statistics will be used to calculate the percentage of days that participants wear the study-issued activity tracker, the percentage of participants who participate in the optional PA video chat support meetings, the percentage of videos viewed by participants, and the percentage of days that daily step goals are achieved.

#### Intervention Uptake

Thematic qualitative analysis [57] will be applied to participant exit interview data to identify barriers and facilitators to participants’ uptake of PACE intervention components.

#### Intervention Effects and Maintenance

Activity tracker data will be used to calculate average steps per week. To explore the intervention effects on outcomes (ie, average weekly steps) from baseline to 3 and 9 months after baseline (6 months after intervention), an intent-to-treat analysis scheme will be adopted, and a mixed-effects model will be fitted to estimate the intervention effect on each outcome. Fixed effects terms will include time point (categorical), intervention group, and intervention × time point, and the model will control for the baseline level of the outcome in addition to age and time since diagnosis. A random intercept will be included to account for within-participant correlation. The estimates and tests of the interaction term will be used to assess the preliminary efficacy of the intervention at each time point. Subgroup analysis by sex will be conducted to explore the difference. Missing data will be carefully imputed using mean or median imputation for continuous variables and mode imputation for categorical variables.

### Dissemination

Study findings will be shared with the research and medical community via conference presentations and peer-reviewed publications. Participants will receive a lay summary of the study findings. At the conclusion of the study, the PACE video library will be made available to the public via the UNC Lineberger Cancer Network video library [58]. The continuation of virtual support sessions will be explored through collaboration with AWF.

## Results

NIMHD funded this study in 2021; NIMHD is not involved in conducting this research. Study enrollment began in August 2024 and is anticipated to conclude in December 2024. As of June 26, 2025, 16 participants have been enrolled.

## Discussion

### Anticipated Findings

The PACE intervention addresses a critical gap in cancer care by focusing on a population that does not engage in recommended levels of PA and that experiences significant disparities in mortality and quality of life—Black survivors of CRC. The PACE study will provide an understanding of how theory-guided intervention components promote PA in this population beyond what is achieved through publicly and widely available trackers and information. Insights on engaging community advisory board members in implementing PA trials will also be provided. The PACE study will evaluate the feasibility of collecting blood samples using an innovative blood spot collection method. This approach has the potential to significantly enhance the feasibility of conducting biomarker research, particularly among hardly reached populations. It eliminates the barriers of participants needing to travel long distances and finding time for laboratory appointments. The findings from the PACE study on blood spot data collection could greatly impact the volume and generalizability of mechanistic exercise oncology research.

The PACE study tests an intervention that was co-designed with African American survivors of cancer. This study will expand our understanding of how co-designed interventions can improve PA and how scientists can better approach designing and implementing future interventions with hardly reached populations. The use of adaptive step goals is responsive to each individual’s circumstances and the evolving needs experienced during cancer treatment. Simultaneously, the PACE intervention is conducive to large-scale implementation, with minimal need for additional interventionist resources. This not only aligns with the increasing role of technology in health care but also provides a scalable and cost-effective method for delivery.

### Limitations and Strengths

Limitations noted in the design of the PACE intervention include the use of self-report measures and the potential for social desirability bias. In addition, depending on how long it takes to obtain consent for participation and set up the activity tracker, participants may begin the PACE intervention at different times after receiving their cancer diagnosis. This may lead to variation in the nature and intensity of side effects participants experience and how effects impact their activity levels. However, the use of a control group, as well as daily step goals that are adaptive to individual participants’ activity levels, will minimize this study’s limitation and allow for the enrollment of participants earlier in the treatment trajectory, as well as an inclusion of a broader range of CRC diagnoses (ie, from early-stage participants undergoing surgery only to those with metastatic disease receiving palliative treatments), all of whom can benefit from PA substantially. Finally, although this study uses previously validated measures, the validity testing for these measures has often been conducted outside the United States, where race and ethnicity are often not reported in study demographics, and much of the research has been conducted with primarily White participants. The PACE study focuses solely on developing and testing an intervention for Black patients because of their historical underrepresentation in prior research. Our study will provide much-needed information on the feasibility of intervention methods and explore intervention effects for this population, including pilot validity data for the exploratory measures used. A further strength of the PACE design is the collaboration with Black survivors of cancer and caregivers in all aspects of this research, from formative research to method development and active engagement in creating and delivering the PACE intervention. Finally, through the use of BCT coding, findings will identify which components of the PACE intervention are most effective and may inform a broad range of future PA interventions for individuals diagnosed with cancer.

### Conclusions

Overall, this research has broad implications for addressing cancer disparities, advancing the understanding of PA’s impact on cancer outcomes, and innovating intervention strategies for populations considered underserved that can be adapted to benefit additional populations.

## Appendix

Multimedia Appendix 1 SPIRIT checklist.

Multimedia Appendix 2 Physical Activity Centers Empowerment intervention components, theoretical constructs targeted, and behavior change techniques used in each component.

## Abbreviations

ART: affective-reflective theory
AWF: Angelic Warrior Foundation
BCT: behavior change technique
CRC: colorectal cancer
DBS: dried blood spot
IL: interleukin
IRB: institutional review board
NIMHD: National Institute for Minority Health and Health Disparities
PA: physical activity
PACE: Physical Activity Centers Empowerment
PAR-Q: Physical Activity Readiness Questionnaire
PROMIS: Patient-Reported Outcomes Measurement Information System
RCA: Rapid Case Ascertainment
RE-AIM: reach, effectiveness, adoption, implementation, and maintenance
REDCap: Research Electronic Data Capture
SHARE: Stiving to Hold Accountability in Research Equity
SPIRIT: Standard Protocol Items: Recommendations for Interventional Trials
UNC: University of North Carolina at Chapel Hill
