# Supplementary material for: Community-Engaged Approach to Increase Physical Activity Among Black Individuals With Colorectal Cancer: Protocol for a Feasibility Randomized Controlled Trial of the Physical Activity Centers Empowerment Study
Source: JMIR Res Protoc. 2025 Oct 17;14:e65804. doi: 10.2196/65804 (PMC12579295; doi:10.2196/65804)
Supplement: Multimedia Appendix 2 [file resprot_v14i1e65804_app2.docx]

Table S1. PACE intervention components, theoretical constructs targeted, and BCTs used in each component.

| Intervention components | Theoretical constructs targeted | BCTs |
| --- | --- | --- |
| Daily adaptive step goal | Knowledge, Self-efficacy | 1.1 Goal setting (behavior), 1.5 Review behavior goal(s) |
| Daily text message with short positive message | Self-efficacy | 3.1 Social support (unspecified) |
| Monthly video chat (Zoom) for PA support | Knowledge,  Outcome expectations,  Self-efficacy,  Habits,  Enjoyment | 3.1 Social support (unspecified) |
| PACE video library topics (intervention delivery day) |  |  |
| Meet John, a cancer survivor (Week 1 Day 7) | Outcome expectations,  Self-efficacy | 3.1 Social support (unspecified), 3.3 Social support (emotional), 5.1 Information about health consequences, 5.6 Information about emotional consequences, 9.1 Credible source |
| Physical activity intensity (Week 2 Day 3) | Knowledge,  Outcome expectations | 4.1. Instruction on how to perform the behavior, 5.1. Information about health consequences, 5.6. Information about emotional consequences, 6.3. Information about others’ approval, 9.1. Credible source, 13.2. Framing/reframing |
| Physical activity safety (Week 2 Day 6) | Knowledge,  Outcome expectations,  Self-efficacy | 1.2. Problem solving, 4.1. Instruction on how to perform the behavior, 4.2. Information about Antecedents, 5.1. Information about health consequences, 5.4. Monitoring of emotional consequences, 5.6. Information about emotional consequences, 6.3. Information about others’ approval, 9.1. Credible source, 15.1. Verbal persuasion about capability, 15.4. Self-talk |
| Meet Karia, an exercise specialist for cancer survivors (Week 3 Day 2) | Outcome expectations,  Self-efficacy | 3.1. Social support (unspecified), 5.1. Information about health consequences, 5.6. Information about emotional consequences, 9.1. Credible source |
| Physical activity benefits during cancer treatments (Week 3 Day 5) | Knowledge,  Outcome expectations,  Self-efficacy,  Enjoyment | 4.1. Instruction on how to perform the behavior, 4.2. Information about Antecedents, 5.1. Information about health consequences, 5.4. Monitoring of emotional consequences, 5.6. Information about emotional consequences, 6.3. Information about others’ approval, 9.1. Credible source, 15.1. Verbal persuasion about capability |
| Physical activity resources for people living beyond cancer (Week 4 Day 1) | Knowledge,  Outcome expectations | 4.1. Instruction on how to perform the behavior, 6.3. Information about others’ approval, 9.1. Credible source |
| Meet Paula, a cancer caregiver (Week 4 Day 4) | Outcome expectations,  Self-efficacy | 5.1. Information about health consequences, 5.6. Information about emotional consequences, 8.7. Graded tasks, 9.1. Credible source, 15.1. Verbal persuasion about capability |
| The right amount of physical activity (Week 4 Day 7) | Knowledge,  Outcome expectations | 1.2 Problem Solving, 3.1. Social support (unspecified), 4.1. Instruction on how to perform the behavior, 5.1. Information about health consequences, 5.6. Information about emotional consequences, 8.7. Graded tasks  9.1. Credible source, 13.2. Framing/reframing, 15.1. Verbal persuasion about capability, 15.4. Self-talk |
| Physical activity and cancer prognosis (Week 5 Day 3) | Knowledge,  Outcome expectations | 5.1. Information about health consequences, 9.1. Credible source, 13.2. Framing/reframing, 15.1. Verbal persuasion about capability |
| Meet Deirdre, a cancer survivor (Week 5 Day 6) | Outcome expectations,  Self-efficacy | 3.1. Social support (unspecified), 5.1. Information about health consequences, 5.6. Information about emotional consequences, 9.1. Credible source, 15.1. Verbal persuasion about capability |
| Talking to your doctor about physical activity (Week 6 Day 2) | Knowledge,  Outcome expectations,  Self-efficacy | 3.1. Social support (unspecified), 5.1. Information about health consequences, 5.6. Information about emotional consequences, 9.1. Credible source, 15.1. Verbal persuasion about capability |
| Physical activity, Energy, Fatigue and Sleep (Week 6 Day 5) | Knowledge,  Outcome expectations,  Self-efficacy | 3.1. Social support (unspecified), 5.1. Information about health consequences, 9.1. Credible source, 15.1. Verbal persuasion about capability |
| Physical activity and social support (Week 7 Day 1) | Self-efficacy,  Habits,  Enjoyment | 1.2 Problem Solving, 3.1. Social support (unspecified), 5.1. Information about health consequences, 6.3. Information about others’ approval, 8.7. Graded tasks, 9.1. Credible source |
| Strength training (Week 7 Day 4) | Knowledge,  Outcome expectations | 4.1. Instruction on how to perform the behavior, 5.1. Information about health consequences, 5.6. Information about emotional consequences, 6.1. Demonstration of the behavior, 6.3. Information about others’ approval, 8.7. Graded tasks, 9.1. Credible source, 15.1. Verbal persuasion about capability |
| Not feeling well enough for physical activity? (Week 7 Day 7) | Knowledge,  Outcome expectations,  Self-efficacy | 3.1. Social support (unspecified), 5.1. Information about health consequences, 8.3. Habit formation, 8.7. Graded tasks, 9.1. Credible source, 13.2. Framing/reframing |
| Diet and hydration for physical activity (Week 8 Day 3) | Knowledge | 4.2. Information about Antecedents, 9.1. Credible source |
| Realistic physical activity goals (Week 8 Day 6) | Self-efficacy,  Habits,  Enjoyment | 1.1. Goal setting (behavior), 1.4. Action planning, 2.3. Self-monitoring of behavior, 3.1. Social support (unspecified), 3.3. Social support (emotional), 5.4. Monitoring of emotional consequences, 7.1. Prompts/cues, 8.3. Habit formation, 8.7. Graded tasks, 9.1. Credible source, 12.1. Restructuring the physical environment, 15.1. Verbal persuasion about capability |
| Enjoying physical activity (Week 9 Day 2) | Self-efficacy,  Habits,  Enjoyment | 3.1. Social support (unspecified), 5.4. Monitoring of emotional consequences, 8.2. Behavior substitution, 9.1. Credible source, 15.1. Verbal persuasion about capability, 15.4. Self-talk |
| Keeping physical activity part of your life (Week 10 Day 1) | Self-efficacy,  Habits,  Enjoyment | 3.1. Social support (unspecified), 7.1. Prompts/cues, 8.2. Behavior substitution, 8.3. Habit formation, 9.1. Credible source, 12.1. Restructuring the physical environment, 15.1. Verbal persuasion about capability |
| SMART physical activity goals (Week 11 Day 1) | Self-efficacy,  Habits  Enjoyment | 1.1. Goal setting (behavior), 2.3. Self-monitoring of behavior, 3.1. Social support (unspecified) |
